# Integrating shear wave elastography into clinical prediction of Graves’ disease recurrence: a novel risk scoring system

**DOI:** 10.3389/fendo.2025.1551983

**Published:** 2025-03-12

**Authors:** Xiao-Yun Zha, Ze-Hong Xu, Jia-Jia Dong, Liang-Xiao Xie, Peng-Bin Lai, Chang-Shun Wei, Hua-Qiang Zheng, Duo-Bin Huang, Jin-Zhi Wu

**Affiliations:** The First Department of Endocrinology and Metabolism, Zhangzhou Affiliated Hospital of Fujian Medical University, Zhangzhou, Fujian, China

**Keywords:** shear wave elastography, Graves’ disease recurrence, risk prediction model, risk scoring system, thyroid stiffness

## Abstract

**Objective:**

This study aims to evaluate the utility of shear wave elastography (SWE) in predicting the recurrence risk of Graves’ disease(GD), to construct a recurrence risk prediction model that integrates SWE and clinical characteristics, and to develop a risk scoring system aimed at enhancing the survival rate of patients with GD following drug treatment and prognosis management.

**Methods:**

A prospective cohort study was conducted involving with 169 patients diagnosed with first-episode GD. By analyzing SWE parameters, three-dimensional thyroid volume, TRAb levels, and other clinical indicators, the Cox proportional hazards model was used to construct a recurrence risk prediction model for GD. Bootstrap resampling was employed to verify the model’s reliability. A simple recurrence risk scoring system was also developed based on independent risk factors for clinical use.

**Results:**

The study identified several factors significantly associated with GD recurrence: age <35 years, a family history of GD, an initial TRAb level≧15 IU/ml, a thyroid volume≧19 cm³, an initial SWE≧2.0 m/s, and a TSH(thyroid stimulating hormone) normalization duration <4 months. Notably, SWE was found to be a strong predictor, with patients exhibiting SWE ≥2.0 m/s having a recurrence risk that is 4.54 times greater than those with lower values. Based on these risk factors, a scoring system was developed with a cutoff of 4 points for recurrence risk, demonstrating a sensitivity of 74% and a specificity of 91.8%. The area under the curve (AUC) of the final model was 0.91, indicating high predictive accuracy.

**Conclusions:**

SWE is an independent predictor of recurrence risk in GD. When combined with traditional clinical indicators, it significantly enhances the predictive capability for GD recurrence. The risk score model provides a simple and effective tool for individualized management and optimization of treatment strategies.

## 1 Introduction

Graves’ disease (GD) is an autoimmune thyroid disorder characterized by hyperthyroidism, goiter, and may also present with ocular symptoms (1). Antithyroid drug (ATD) treatment is the first-line approach for managing GD; however, the recurrence rate following treatment remains high, posing a challenge for the long-term management of affected patients (2). Accurate identification of individuals at high risk for recurrence is crucial for optimizing treatment strategies and enhancing patient outcomes. Current studies highlight the multifactorial nature of GD recurrence, identifying age, family history, smoking history, goiter size, and initial TRAb (thyrotropin receptor antibody) levels as well-established predictors (2–5). However, individual risk factors exhibit limited reliability and applicability when assessing the likelihood of GD recurrence following ATD treatment. A multifactorial prediction model can assist newly diagnosed patients in selecting the most appropriate treatment plan, thereby facilitating more precise treatment and enhancing the GD management. In recent years, researchers have developed several prediction models, including the GREAT score, GREAT+ score, and clinical severity score (CSS). The development and ongoing research of these models offer new tools for assessing the recurrence risk associated with GD (6–8).

Despite advances in understanding the risk factors for the recurrence of GD, gaps remain in integrating novel diagnostic approaches. Among these, SWE has emerged as a promising non-invasive technique that may provide additional insights into recurrence risk by assessing the mechanical properties of thyroid tissue, particularly the degree of fibrosis. In the field of thyroid disease diagnosis and treatment, ultrasound elastography technology has gradually gained attention due to its non-invasive, real-time, and highly reproducible characteristics (9). Studies have demonstrated that thyroid ultrasound elastography offers unique advantages in distinguishing between benign and malignant thyroid nodules, diagnosing and evaluating the nature and activity of thyroid diseases, and reflecting the pathophysiological changes of thyroid tissue to a certain extent (10, 11). In recent years, numerous studies have confirmed the value of ultrasound elastography in diagnosing thyroid diseases. According to Nattabi et al., the sensitivity of ultrasound elastography in differentiating benign from malignant thyroid nodules can reach 66%, with a specificity of 78% (12). Furthermore, the research conducted by Li et al. found that the ultrasound elastography score is closely associated with disease activity in patients with hyperthyroidism, providing compelling evidence for its use in managing GD (13). This technique may also predict the recurrence of GD by evaluating the elastic characteristics of thyroid tissue. Given that the degree of inflammation and fibrosis in thyroid tissue may correlate with the risk of disease recurrence, the tissue stiffness information provided by ultrasound elastography could serve as a crucial indicator for predicting such recurrences. However, there is currently no research investigating the use of thyroid ultrasound elastography in predicting the recurrence risk of GD, which presents new avenues for exploring the application of ultrasound elastography in developing a recurrence risk model for GD.

Given the high risk of recurrence of GD following drug treatment, coupled with the potential of ultrasound elastography technology in the diagnosis and management of thyroid disorders, this study aimed to develop a recurrence risk model for GD based on ultrasound elastography. A prospective cohort study design was employed, including a group of patients with GD undergoing ATD treatment. By analyzing their clinical data, thyroid ultrasound characteristics, and treatment responses, the study investigated the correlation between ultrasound elastography parameters and the risk of GD recurrence. The development of a risk prediction model that integrates ultrasound elastography parameters with clinical characteristics (such as medical history and laboratory results) provides clinicians with a straightforward and effective predictive tool to assist in optimizing treatment plans and minimizing recurrence risk. Furthermore, this study aimed to establish a new theoretical foundation for the application of ultrasound elastography technology within the field of thyroid diseases.

## 2 Materials and methods

### 2.1 Study subjects

A total of 351 patients with first-episode GD who received ATD treatment were followed up at the Department of Endocrinology and Metabolism at Zhangzhou Hospital, affiliated with Fujian Medical University, from 2021 to 2022. As of June 2024, 169 patients had completed the 2-year follow-up after drug withdrawal and were included in the study. A model development cohort was established, with bootstrap resampled data used as a model validation cohort within the same dataset. Among the 182 patients who were not enrolled in the study, 73 were lost to follow-up, 4 underwent surgery, 58 received radioactive iodine (RAI) treatment, and 47 discontinued treatment and were followed for less than 2 years. Patients were categorized into a recurrence group and a remission group based on whether they experienced a recurrence within 2 years after discontinuing the medication.

The diagnosis of Graves’ disease (GD) in this study was based on clinical features, laboratory tests, and imaging findings, in accordance with the guidelines from the American Thyroid Association (ATA) (14) and the European Thyroid Association (ETA) (15). The diagnostic criteria included: (1) Clinical Features: Symptoms such as hyperthyroidism, goiter, and ophthalmopathy. (2) Laboratory Tests: A reduced TSH level (<0.270 mIU/L) and elevated free thyroxine (FT4) levels (>22.0 pmol/L). (3) Thyroid-Stimulating Hormone Receptor Antibody (TRAb): A positive TRAb level (>1.5 IU/L). (4) Imaging: Radioactive iodine uptake or findings from thyroid Doppler ultrasonography. It is important to note that not all of the above criteria need to be met for a diagnosis of GD. For instance, the presence of clinical features, along with biochemical tests showing reduced TSH and elevated FT4, or a positive TRAb test, could be sufficient. All patients included in this study tested positive for TRAb, which strongly supports the diagnosis of GD.

Inclusion criteria: (1) Individuals aged 18 to 80 years; (2) Patients newly diagnosed with GD; (3) Patients not currently receiving any ATD treatment. Exclusion criteria: (1) Presence of primary diseases such as cardiovascular or cerebrovascular conditions, liver or kidney disorders, hematopoietic system diseases, tumors, or mental illnesses; (2) Severe complications associated with hyperthyroidism, including but not limited to severe liver dysfunction (defined as aminotransferase levels exceeding three times the upper limit), blood neutrophil counts below 1×10^9/L, malignant arrhythmias, heart failure, or cardiac ejection fractions below 50%, and hyperthyroid crises; (3) Serious adverse reactions to ATDs during treatment, such as allergies, drug-induced liver damage, or agranulocytosis; (4) Patients exhibiting poor compliance who request to withdraw from follow-up or are lost to follow-up; (5) Patients who switch to radioactive iodine or surgical treatment during ATD therapy.

The baseline data collected for the enrolled population included general information such as age, gender, smoking history, and family history. Auxiliary examinations comprised levels of free triiodothyronine (FT3), FT4, TSH, TRAb, thyroid peroxidase autoantibody(TPOAb), and thyroglobulin autoantibody(TGAb), as well as ultrasound measurements of three-dimensional thyroid volume, thyroid gland shear wave elastic velocity, and peak velocity of superior thyroid artery flow (STA-PV). The initial doses of ATDs were methimazole (MMI) at 10 to 30 mg/day and propylthiouracil (PTU) at 150 to 300 mg/day. Following improvements of symptoms and thyroid function, the maintenance doses of MMI were gradually reduced to 2.5 mg/day and PTU to 25 mg/day, with further adjustments based on the patient’s condition. Regular follow-up reminders were provided via telephone, and data (including thyroid function, thyroid autoantibodies, and drug dosage) were recorded on a standardized paper form during each follow-up visit. Assessments were conducted monthly for the first six months and then every two months until the discontinuation criteria were met. Recurrence was evaluated at 1, 3, 6, 12, 18, and 24 months after the discontinuation of treatment.

Criteria for discontinuation include achieving normal serum levels of FT3, FT4, and TSH following standard treatment with ATD, alongside a reduction in TRAb to normal levels. The dosage should then be gradually decreased to a low-dose ATD (e.g., MMI ≤ 2.5 mg/day) to maintain normal thyroid function and ensure TRAb remains negative for at least six months. Prognostic criteria are as follows: (1) Remission is defined as FT3, FT4, and TSH all remaining within the normal range for two years post-ATD discontinuation; (2) Relapse is indicated by an increase in FT3 and/or FT4 levels and a decrease in TSH during follow-up after ATD has been discontinued. This study received approval from the Ethics Committee of Zhangzhou Hospital Affiliated to Fujian Medical University, and all research participants provided informed consent.

### 2.2 Methods

#### 2.2.1 Measurement of serum levels

Serum levels of TSH, FT4, FT3,TRAb, and TPOAb were measured using an electrochemiluminescence immunoassay (Beckman, DXI800, USA) in venous blood samples collected from patients after overnight fasting. The normal ranges for FT3, FT4, TSH, TRAb, TPOAb, and TGAb are as follows: FT3: 3.10–6.80 pmol/L; FT4: 7.5–21.1 pmol/L; TSH: 0.34–5.6 µIU/mL; TRAb: <1.75 IU/L; TPOAb: <9 IU/L; and TGAb: <34 IU/L.

#### 2.2.2 Thyroid volume and blood flow assessment

The three-dimensional thyroid volume and STA-PV were assessed using the GELOGIQ E8 ultrasonic diagnostic instrument with a three-dimensional volume probe (RAB-6). The thyroid was scanned transversely from top to bottom, and the largest transverse section was selected and stabilized with the probe. The three-dimensional ultrasonic volume automatic measurement technology was then initiated to collect and store images of the thyroid. The equipment automatically reconstructed a simulation model of the thyroid and calculated its volume (cm³). Blood flow distribution within the thyroid was evaluated using color Doppler blood flow imaging, while pulsed Doppler was employed to determine the patient’s STA-PV level.

#### 2.2.3 Shear wave elastography assessment

**Figure 1:**
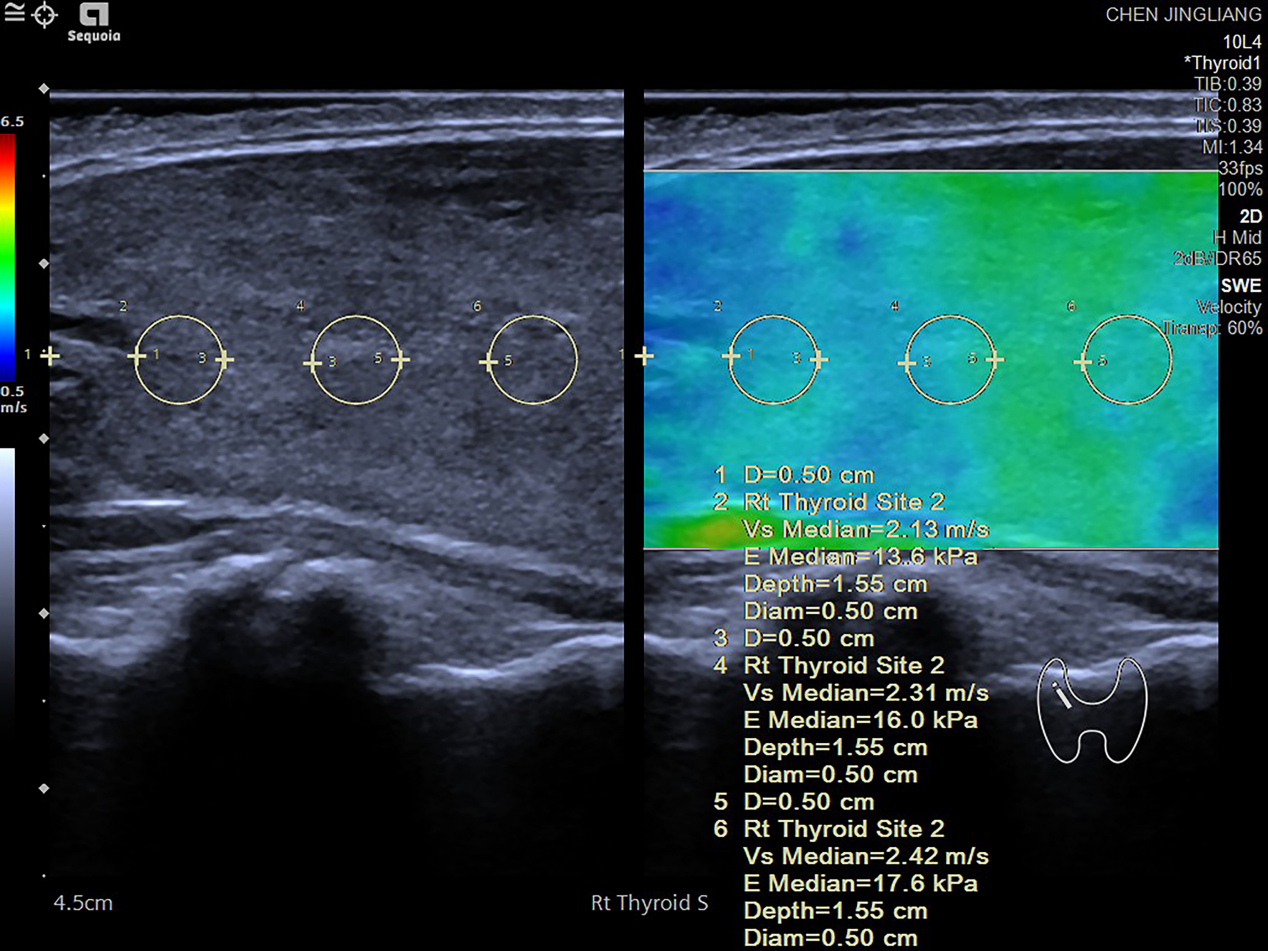
Shear wave elastography (SWE) measurement process conducted on various regions of the thyroid gland. The measurement begins 5 mm below one side of the thyroid pole, with a region of interest (ROI) set at 3 mm. Measurements are taken at intervals of 5 mm, with three measurements recorded on each side. The shear wave speed (in m/s) and the corresponding elastic value (in kPa) are documented. The final value for each side is computed as the average of the three measurements. Elevated shear wave speed and high kPa values typically indicate tissue hardening, which may suggest the presence of fibrosis or abnormal tissue changes. The accompanying color diagram reflects the hardness of the thyroid tissue, where deeper colors correspond to greater hardness.

The shear wave elastic velocity of the thyroid gland was measured using the SEMENSACUSON Sequoia 512 ultrasonic diagnostic instrument and a 10L4 phased array probe. In elastography mode, the largest longitudinal section of the thyroid gland on one side was selected, ensuring that blood vessels and nodules were avoided. After stabilizing the image, the frame was frozen, and measurements commenced from the center of the gland, 5 mm away from the upper pole, with measurements taken every 5 mm for a total of three measurements. The average of these measurements represented the shear wave elastic velocity of the thyroid gland on that side. This process was then repeated for the other side of the thyroid gland, with the bilateral average value calculated to determine the overall shear wave elastic velocity of the patient’s thyroid gland (**Figure 1**).

### 2.3 Statistical analysis

Statistical analysis was conducted using R version 4.0.5 (http://www.rproject.org/). After performing the Kolmogorov-Smirnov normality test, continuous variables that followed a normal distribution are expressed as “mean ± standard deviation,” with comparisons between groups conducted using one-way analysis of variance. For continuous variables that did not follow a normal distribution, data are presented as “Median [Q25, Q75],” and comparisons between groups are conducted using the Wilcoxon rank sum test. Categorical variables are presented as “cases (rates),” and the chi-square test is employed for group comparisons. The median value of a continuous variable serves as the cutoff point. This study utilizes the entire cohort dataset as the discovery cohort, with bootstrap resampled data is employed as the validation cohort. The Cox proportional hazards model was employed to evaluate prognostic factors for GD recurrence. Final variables were selected based on statistical results to construct a GD recurrence risk prediction model. The relative risk of GD recurrence was calculated using the hazard ratio (HR) and its 95% confidence interval (CI). Additionally, a calibration plot curve was generated, and the Brier score was calculated to assess model calibration. In addition, a scoring system was developed to simplify the calculation of relapse risk following the discontinuation of ATD. The scores were derived from the HR coefficients of the predictors identified in the final model. To calculate a score for each risk factor, the regression coefficient (logarithm of HR) for that variable was divided by the lowest value of the regression coefficient, with the result rounded accordingly. Independent risk factors such as age <35 years, thyroid volume ≧19 cm³, and TSH normalization duration less than 4 months were assigned 1 point. A family history of GD and an initial TRAb level ≧15 IU/ml were assigned 1.5 points, while an initial SWE of ≧2.0 m/s, considered an independent risk factor, was assigned 2 points, resulting in a maximum total score of 8 points. Each individual risk score was used as the test variable, with actual follow-up results serving as the state variable to construct a receiver operating characteristic (ROC) curve. This approach allowed for the identification of the maximum value of the Youden index and determination of the optimal risk stratification threshold of 4 points. Patients were classified according to this risk score stratification threshold. The Kaplan-Meier method was employed to estimate the cumulative remission rate for each risk group over a 2-year period, and the log-rank test was used for comparative analysis. A p-value of <0.05 was considered statistically significant.

## 3 Results

### 3.1 Clinical features of patients with Graves’ disease between recurrence and remission groups

**Table 1** presents the clinical characteristics of patients with GD categorized by their relapsing and remitting status. A total of 169 patients were included in this study, with a median age of 35 years and 32.54% being male. The remission group consisted of 73 patients (43.2%), while the recurrence group included 96 patients (56.8%). There were no statistically significant differences between the two groups regarding gender, initial FT3/FT4 ratio, FT3, FT4, TPOAb, TGAb levels, STA-PV, MMDT duration, TSH maintenance level, and TRAb level at discontinuation. However, the recurrence group exhibited significantly higher values than the remission group for age, family history of GD, smoking status, initial TRAb, thyroid three-dimensional volume, and SWE (P < 0.05). Additionally, “TSH normalization duration”, which refers specifically to the duration during treatment in which TSH levels remain within the normal range, was found to be shorter in the recurrence group compared to the remission group (P < 0.05).

**Table 1:** Clinical features of patients with Graves’ hyperthyroidism between recurrence and remission groups [Mean ± SD,M (Q1,Q3)].

Variables	Total (n = 169)	Remission (n = 73)	Recurrence (n = 96)	Statistic	*P*
AGE(year)	35.00 (30.00, 42.00)	40.00 (32.00, 48.00)	33.50 (29.00, 38.00)	Z=-4.63	<0.001
Gender, n(%)				χ²=2.30	0.129
Male	55 (32.54)	29 (38.67)	26 (27.66)		
Female	114 (67.46)	46 (61.33)	68 (72.34)		
Smoking, n(%)				χ²=6.01	0.014
None	120 (71.01)	59 (80.82)	61 (63.54)		
Ex- or current	49 (28.99)	14 (19.18)	35 (36.46)		
Family history of GD, n(%)				χ²=15.94	<0.001
NO	86 (50.89)	50 (68.49)	36 (37.50)		
YES	83 (49.11)	23 (31.51)	60 (62.50)		
Initial FT3(pmol/l)	19.71 ± 10.12	19.61 ± 10.35	19.80 ± 9.99	t=-0.12	0.904
Initial FT4,(pmol/l)	50.81 ± 16.48	50.16 ± 14.42	51.31 ± 17.95	t=-0.45	0.655
Initial FT3/FT4	0.38 ± 0.10	0.37 ± 0.10	0.38 ± 0.09	t=-0.16	0.872
Initial TRAb(IU/ml)	14.84 ± 8.62	10.77 ± 6.73	17.94 ± 8.65	t=-6.05	<0.001
TRAb at withdraw (IU/ml)	0.98 ± 0.31	0.98 ± 0.31	0.99 ± 0.30	t=-0.18	0.86
Initial TPOAb (IU/ml)	261.56 ± 374.19	291.32 ± 416.55	238.93 ± 338.98	t=0.90	0.369
Initial TGAb (IU/ml)	76.39 ± 195.29	71.14 ± 154.25	80.34 ± 222.06	t=-0.30	0.765
Initial STA-PV (m/s)	0.65 ± 0.30	0.64 ± 0.29	0.66 ± 0.32	t=-0.38	0.707
Initial Thyroid volume(cm3)	18.90 (14.80, 25.00)	16.70 (14.00, 20.00)	21.20 (16.50, 28.97)	Z=-3.95	<0.001
Initial SWE(m/s)	2.04 (1.62, 2.42)	1.64 (1.44, 1.87)	2.33 (2.04, 2.69)	Z=-7.82	<0.001
Duration of MMDT(month)	5.00 (4.00, 7.00)	5.00 (3.00, 7.00)	5.00 (4.00, 7.00)	Z=-1.11	0.267
TSH normalization duration, n(%)				χ²=10.81	0.001
<4month	87 (51.48)	27 (36.99)	60 (62.50)		
≧4month	82 (48.52)	46 (63.01)	36 (37.50)		

t, t-test; Z, Mann-Whitney test; χ², Chi-square test; SD, standard deviation; M, Median; Q1, 1st Quartile; Q3, 3st Quartile; GD, Graves’ disease; FT3, free triiodothyronine; FT4, free thyroxine; TRAb, thyrotropin receptor antibody; TPOAb, thyroid peroxidase autoantibody; TgAb, thyroglobulin autoantibody; TSH, thyroid stimulating hormone; SWE, shear wave elastography; STA-PV, Peak velocity of superior thyroid artery flow; MMDT, minimum maintenance dose therapy.

The median value of a continuous variable serves as the cutoff point.

### 3.2 Risk factors associated with recurrence of Graves’ disease

The results of the univariate Cox regression analysis (**Table 2**) indicated that several factors are associated with an increased risk of GD recurrence in patients. Specifically, individuals younger than 35 years (HR = 3.00, 95% CI: 1.75–5.14, P < 0.001), those with a history of smoking (HR = 1.66, 95% CI: 0.99–2.78, P = 0.046), a family history of GD (HR = 2.88, 95% CI: 1.67–4.96, P < 0.001), initial TRAb levels≧15 IU/ml (HR = 3.14, 95% CI: 1.85–5.35, P < 0.001), initial thyroid volumes≧19 cm³ (HR = 2.08, 95% CI: 1.22–3.54, P = 0.007), initial SWE values ≧ 2.0 m/s (HR = 5.33, 95% CI: 2.89–9.81, P < 0.001), and TSH normalization duration less than 4 months (HR = 1.95, 95% CI: 1.14–3.32, P = 0.014) were identified as significant risk factors. The cutoff values for age, initial TRAb level, thyroid volume, SWE, and TSH normalization duration were determined based on the median values across all patients. The relationship between gender, TSH maintenance levels, and recurrence risk was found to be insignificant. These results suggest that smoking status, family history, younger age, higher initial TRAb levels, thyroid volume, and SWE may serve as potential risk factors for the recurrence of GD.

**Table 2:** Univariate cox regression analysis to predict the risk factors of recurrence of Graves’ disease.

Variables	β	*P*	HR (95%CI)
Age			
≧35year			1.00 (Reference)
<35year	1.1	<0.001	3.00 (1.75 ~ 5.14)
Gender			
Male			1.00 (Reference)
Female	-0.47	0.086	0.63 (0.37 ~ 1.07)
Smoking			
None			1.00 (Reference)
Ex- or current	0.51	0.046	1.66 (1.02 ~ 2.78)
Family history of GD			
NO			1.00 (Reference)
YES	1.06	<.001	2.88 (1.67 ~ 4.96)
Initial TRAb			
<15 (IU/ml)			1.00 (Reference)
≧15 (IU/ml)	1.15	<0.001	3.14 (1.85 ~ 5.35)
Initial Thyroid volume			
<19 (cm3)			1.00 (Reference)
≧19 (cm3)	0.73	0.007	2.08 (1.22 ~ 3.54)
Initial SWE			
<2.0 (m/s)			1.00 (Reference)
≧2.0 (m/s)	1.67	<0.001	5.33 (2.89 ~ 9.81)
TSH maintenance levels			
<1 (uIU/ml)			1.00 (Reference)
1-3 (uIU/ml)	-0.43	0.177	0.65 (0.35 ~ 1.21)
3-6 (uIU/ml)	-0.11	0.724	0.90 (0.49 ~ 1.65)
TSH normalization duration			
≧4month			1.00 (Reference)
<4month	0.67	0.014	1.95 (1.14 ~ 3.32)

HR, Hazards Ratio; CI, Confidence Interval; GD, Graves’ disease; TRAb, thyrotropin receptor antibody; TSH, thyroid stimulating hormone; SWE, Shear wave elastography.

### 3.3 Correlation between SWE and recurrence risk of Graves’ disease

In a multivariable analysis of the risk of GD recurrence, we evaluated the impact of initial SWE on the recurrence of GD, both with and without adjustments for other potential risk factors (**Table 3**). In the original analysis, which did not account for additional factors, patients with an SWE of 2.0 m/s or greater exhibited a significantly higher risk of recurrence (HR = 5.33, 95% CI: 2.89–9.81, P < 0.001). In Model A, after adjusting for age, gender, family history, smoking history, and TRAb levels, patients with elevated SWE values still demonstrated a significantly increased risk of recurrence (HR = 4.79, 95% CI: 2.65–8.65, P < 0.001). This indicates that elevated SWE remains an independent risk factor for GD recurrence, even after considering other clinically relevant factors. Furthermore, in Model B, after additional adjustments for three-dimensional thyroid volume and TSH normalization duration, the elevated risk of recurrence in patients with high SWE values remained significant (HR = 4.54, 95% CI: 2.46–8.37, P < 0.001), thereby reinforcing the importance of SWE as a risk factor for recurrence. In summary, the increase in SWE value is a significant predictor of recurrence risk in patients with GD. This effect remains significant even after adjusting for other potential influencing factors, indicating that clinicians should prioritize the measurement of SWE values in practice. Consequently, treatment strategies may need to be modified based on this indicator to mitigate the risk of recurrence.

**Table 3:** Hazard ratios of TRAb patterns for recurrence-free survival using multivariate analysis.

Models	HR (95% CI)	P value
Unadjusted		
Initial SWE		
<2.0 (m/s)	1.00 (Reference)	
≧2.0 (m/s)	5.33 (2.89 ~ 9.81)	<0.001
Adjusted model A		
Initial SWE		
<2.0 (m/s)	1.00 (Reference)	
≧2.0 (m/s)	4.79 (2.65 ~ 8.65)	<0.001
Adjusted model B		
Initial SWE		
<2.0 (m/s)	1.00 (Reference)	
≧2.0 (m/s)	4.54 (2.46 ~ 8.37)	<0.001

SWE, Shear wave elastography; HR, hazard ratio; CI, confidence interval.

Adjusted model A was controlled for age, sex, family history of GD, smoking history, and TRAb levels. Adjusted model B was controlled for age, sex, family history of GD, smoking history, TRAb levels, three-dimensional thyroid volume, and TSH normalization duration.

### 3.4 Establishment of recurrence risk prediction model for Graves’ disease

**Figure 2:**
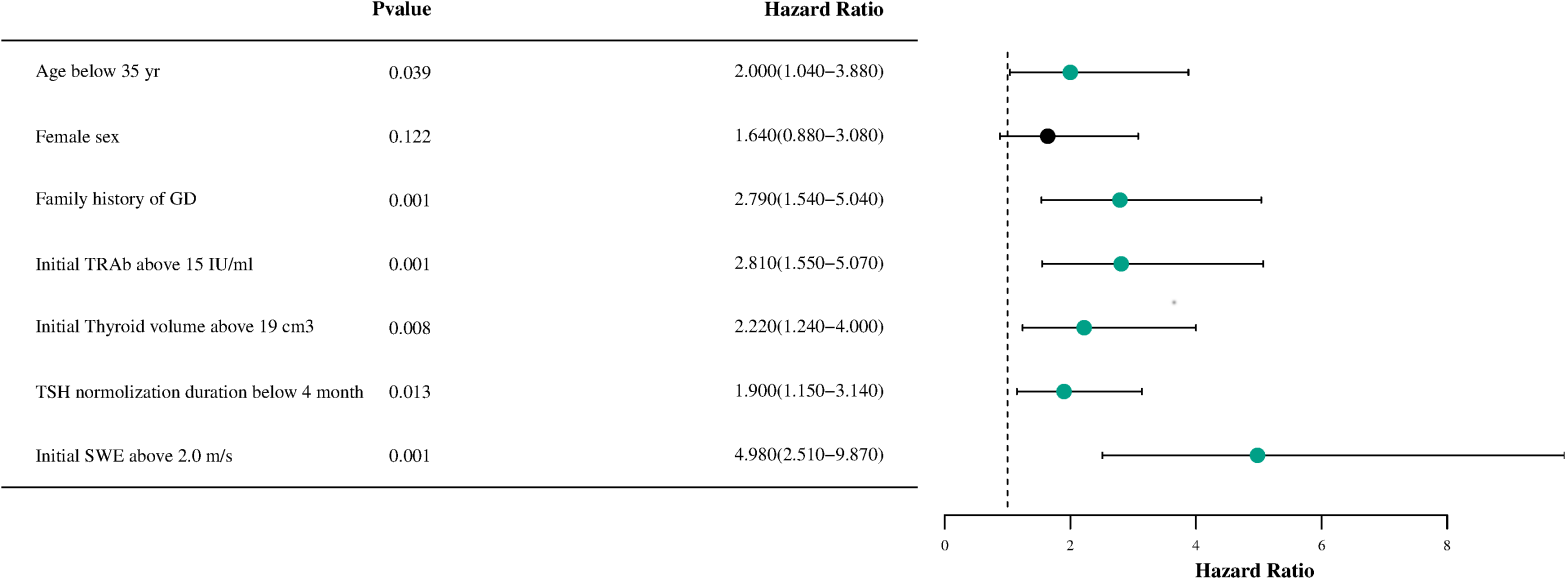
A forest plot of the Cox proportional hazards model for recurrence-free survival in patients with Graves’disease is shown. HR, hazard ratio; GD, Graves' disease; TRAb, thyrotropin receptor antibody; TSH, thyroid stimulating hormone; SWE, Shear wave elastography.

To identify the predictive factors included in the model, a Cox multifactor stepwise regression analysis was conducted, with the recurrence of GD following drug withdrawal serving as the dependent variable. The aforementioned risk factors related to recurrence were treated as independent variables. **Figure 2** illustrates the results of the multivariable Cox regression analysis for GD, including a forest plot depicting the relapse risk. The analysis results indicated that several factors are independent risk factors for the recurrence of GD. These factors include being younger than 35 years old (HR, 2.0; 95% CI, 1.04–3.88; P=0.039), having a family history of GD (HR, 2.79; 95% CI, 1.54–5.04; P<0.001), an initial TRAb level ≧15 IU/ml (HR, 2.81; 95% CI, 1.55–5.07; P<0.001), an initial thyroid volume ≧19 cm³ (HR, 2.22; 95% CI, 1.24–4.00; P=0.008), a TSH normalization duration of less than 4 months (HR, 1.90; 95% CI, 1.15–3.14; P=0.013), and an initial SWE of ≧2.0 m/s (HR, 4.98; 95% CI, 2.51–9.87; P<0.001). However, gender was not found to be an independent risk factor for the recurrence of GD (P =0.122).

### 3.5 Evaluation of Graves’ disease recurrence prediction model

**Figure 3:**
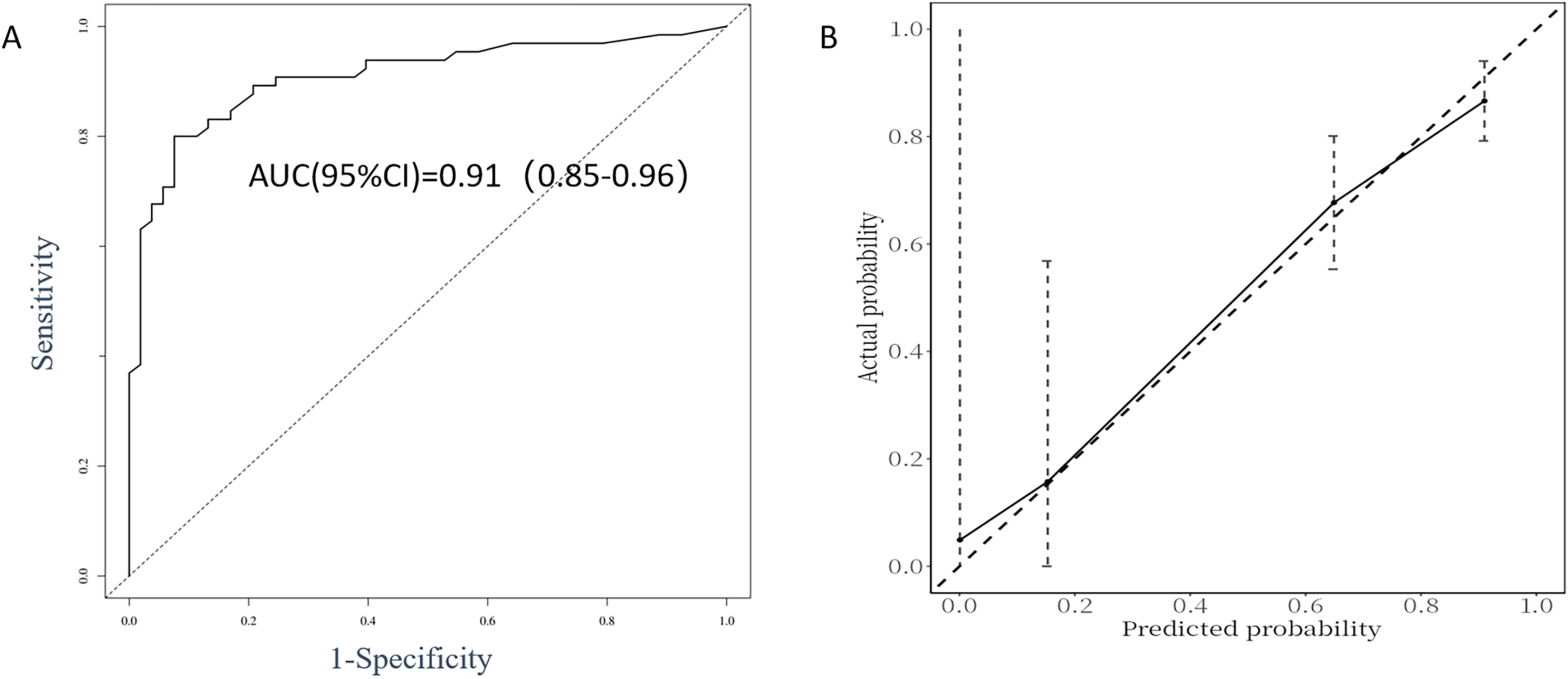
ROC curve analysis **(A)** and calibration plot **(B)** for the predictive power of the proposed predictors in the Cox proportional model.

The performance evaluation of the GD recurrence prediction model primarily involves two key indicators: discrimination and calibration. By plotting the ROC curve (**Figure 3A**) and the calibration curve (**Figure 3B**) using R, we obtained an AUC value of 0.91, with a 95% CI ranging from 0.85 to 0.96. This indicates that the model is effective at distinguishing between relapsed and non-relapsed patients, demonstrating high accuracy. The C-index is 0.87, further confirming the model’s discriminative ability. In the calibration curve (**Figure 3B**), the reference line closely aligns with the fitting curve, indicating a high level of consistency between the model’s predictions and actual observations. Additionally, the Brier score was calculated to be 0.126, significantly lower than the threshold of 0.25, which suggests that the model is well calibrated; that is, the probabilities predicted by the model are very close to the actual probabilities of occurrence. Based on these results, we conclude that the GD recurrence prediction model performs well in terms of both discrimination and calibration, making it an effective predictive tool.

### 3.6 Risk score of Graves’ disease recurrence model

**Figure 4:**
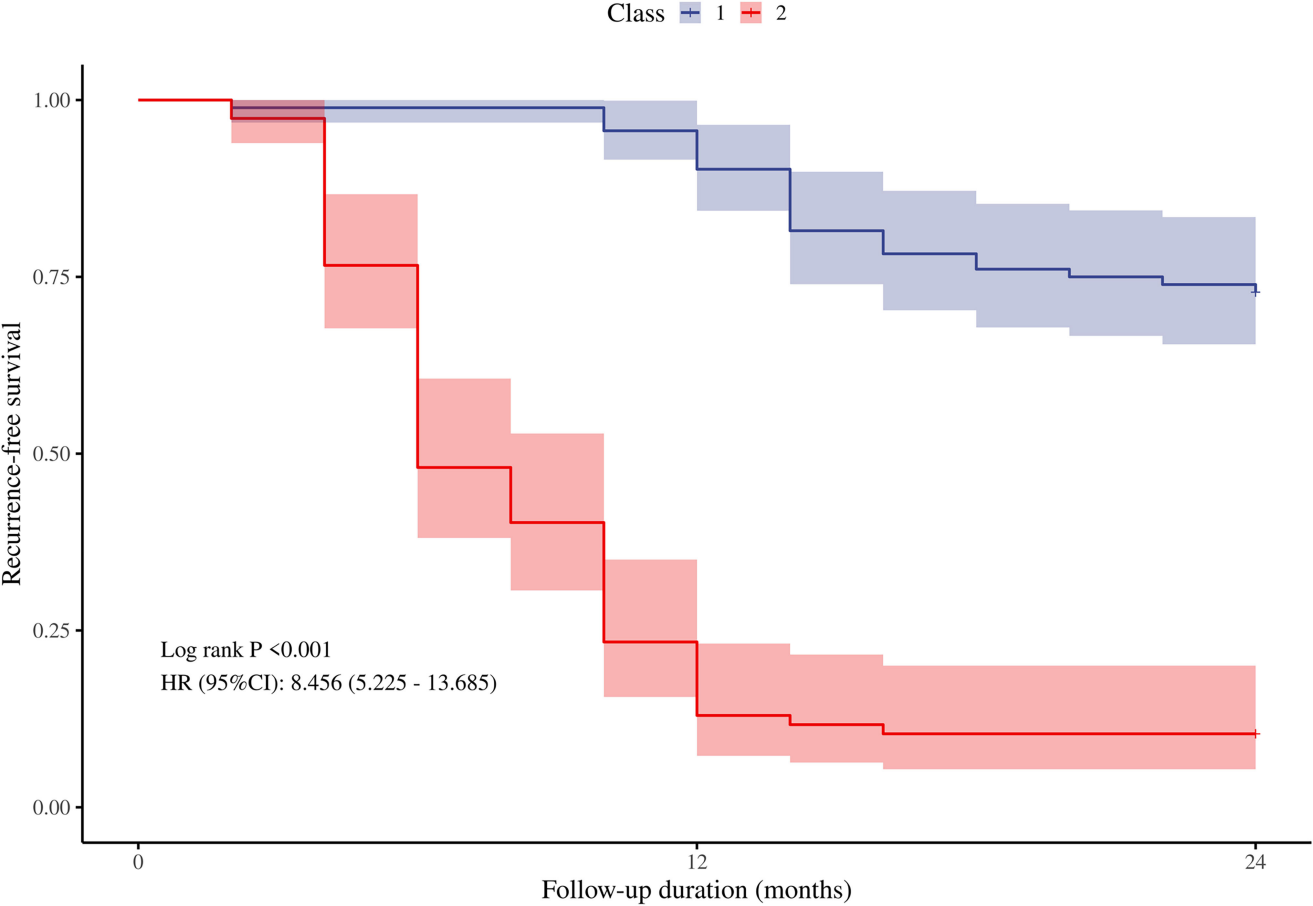
The cumulative recurrence-free survival rate of the two groups within 2 years after drug withdrawal was compared according to the cut-off value of the risk score. Classs1, risk score<4 points; Class2, risk score≧4 points.

As described in the methods section, we developed a recurrence risk prediction model for GD using multivariate analysis. To enhance clinical applicability, we assigned a risk score to each variable, thereby quantifying the contribution of each factor to the overall risk (see **Table 4**). The scoring method outlined in the table involves dividing the regression coefficient of each variable (the logarithm of the hazard ratio) by the smallest regression coefficient and subsequently rounding the result to derive the score. In this model, independent risk factors such as age < 35 years, thyroid volume ≧19 cm³, and TSH normalization duration of less than 4 months were assigned 1 point each. Additionally, having a family history of GD and an initial TRAb level ≧15 IU/ml were assigned 1.5 points. An initial SWE of ≧2.0 m/s is considered an independent risk factor and is assigned 2 points, leading to a maximum total score of 8 points. Using each individual risk score as the test variable and the actual follow-up results as the state variable, the ROC curve calculation yielded an AUC of 0.878 (95% CI = 0.828-0.931, standard error = 0.027, p < 0.01). The maximum value of the Youden index was identified, establishing a risk stratification threshold of 4 points, which corresponds to a sensitivity of 74% and a specificity of 91.8%. When the risk score is ≧4, the likelihood of GD recurrence within 2 years is significantly elevated. Patients were classified according to this risk score stratification threshold, and a Kaplan-Meier curve (**Figure 4**) was generated. The survival curve demonstrated a well-distributed pattern, indicating that the cumulative remission rate of patients within 2 years gradually decreases as follow-up time extends post-drug withdrawal. A log-rank test was employed to compare the cumulative recurrence rates between the two groups over the 2-year period. The results indicated that the recurrence rate for patients with a risk score of 4 or greater (89.6%) was significantly higher than that for those with a risk score of less than 4 (27.2%) (HR = 8.456, 95% CI = 5.225-13.685, p < 0.05). This grading system assists clinicians in assessing a patient’s risk of recurrence and formulating a personalized monitoring and treatment plan.

**Table 4:** Risk score and model for predicting the recurrence of Graves’ hyperthyroidism.

Risk factor	Score
Age	
≧35year	0
<35year	1
Family history of GD	
NO	0
YES	1.5
Initial TRAb	
<15 (IU/ml)	0
≧15 (IU/ml)	1.5
Initial Thyroid volume	
<19 (cm3)	0
≧19 (cm3)	1
Initial SWE	
<2.0 (m/s)	0
≧2.0 (m/s)	2
TSH normalization duration	
≧4month	0
<4month	1
Total	8 points
Class	Class1: 0–3.5 points
	Class2: 4–8 points

GD, Graves’ disease; TRAb, thyrotropin receptor antibody; TSH, thyroid stimulating hormone; SWE, Shear wave elastography.

### 3.7 Validation of the Graves’ recurrence risk score model

**Figure 5:**
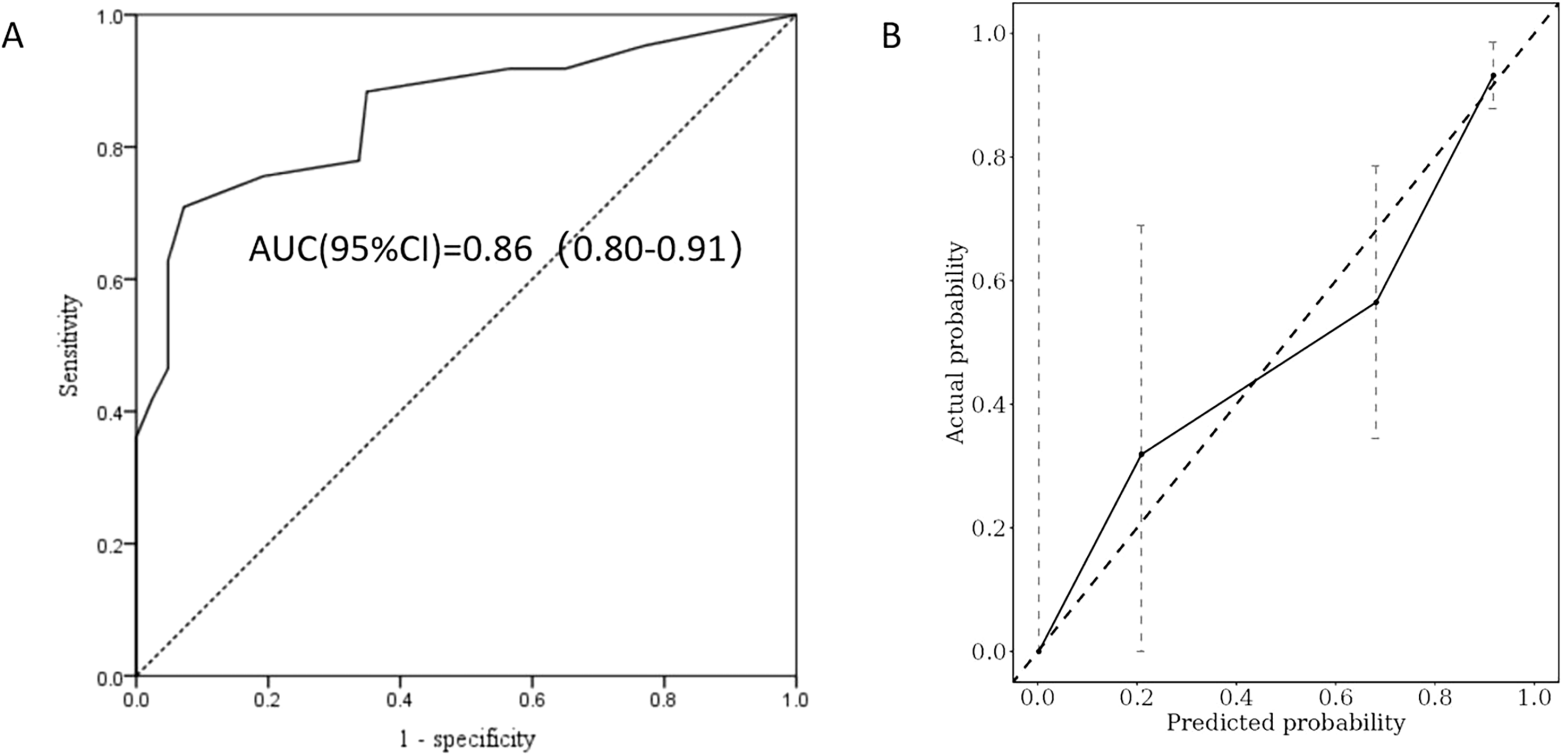
ROC curve analysis **(A)** and calibration plot **(B)** were used to evaluate the predictive power of the recurrence risk score model in the validation cohort.

Bootstrap resampling data was utilized as the validation cohort for the internal validation of the recurrence risk score model. A total of 169 patients were included in this cohort, with a median age of 36 years and 39.1% of the participants being male. The remission and recurrence groups comprised 83 (49.1%) and 86 (50.9%) patients, respectively. The calculated AUC in the validation cohort was 0.86 (95% CI: 0.80–0.91) (**Figure 5A**), with an adjusted C-index of 0.82. The calibration curve (**Figure 5B**) indicates that the actual recurrence event curve closely aligns with the ideal prediction line, demonstrating that the overall predicted recurrence rate is near the actual observed value, and the adjusted Brier score is 0.171. These results suggest that the new model exhibits good discrimination and calibration for assessing the risk of GD recurrence within two years following drug discontinuation in the internal validation cohort.

## 4 Discussion

In this study, we developed a model to predict the risk of GD recurrence by analyzing the clinical characteristics and thyroid ultrasound elastography parameters of affected patients. The results indicated that several factors, including age younger than 35 years, a history of smoking, a family history of GD, high initial TRAb levels (≧15 IU/ml), large thyroid volume (≧19 cm³), shear wave elastic velocity (SWE ≧2 m/s), and TSH normalization duration (<4 months), were significantly associated with the risk of recurrence. Notably, an increase in SWE value (≧2.0 m/s) emerged as an independent predictor of recurrence risk, significantly enhancing the model’s predictive capability even after adjusting for other clinical factors. This study is the first to incorporate SWE into the prediction model for GD recurrence risk, highlighting its unique role in this context. In comparison to traditional predictive indicators such as TRAb and thyroid volume, SWE more directly reflects the stiffness and degree of fibrosis in thyroid tissue, potentially providing a more accurate assessment of recurrence risk. These findings suggest that integrating SWE into clinical practice could facilitate more individualized management strategies, ultimately improving patient outcomes by identifying those at higher risk of recurrence.

GD is the most prevalent cause of hyperthyroidism, primarily treated with ATDs in China, Japan, and Europe, while RAI is the preferred treatment option in the United States (16). In our study population, the recurrence rate of GD within two years following the discontinuation of ATD treatment was found to be 56.8%, aligning with findings from previous reports (17, 18). Traditional predictors of recurrence risk have been identified in multiple studies, highlighting family history, smoking, thyroid volume, Graves’ ophthalmopathy, and TRAb levels as significant indicators (17, 19–21). Our results corroborate these studies, particularly regarding the importance of family history, TRAb levels, and thyroid volume as predictors. Notably, we found that the TSH normalization duration also significantly influences the risk of recurrence. Specifically, our study found that a longer duration of TSH normalization during treatment is typically associated with more stable thyroid function and a lower risk of recurrence. After reviewing the available literature, we found that while there is substantial evidence supporting the association between TSH levels and the risk of recurrence in GD patients, specific studies directly addressing the duration of TSH normalization and its impact on remission stability are limited. The meta-analysis conducted by Subekti et al. demonstrated that patients with suppressed TSH levels have a higher relapse risk compared to those with normal TSH levels (22). Furthermore, a large-scale cohort study confirmed that delayed TSH normalization is associated with an increased risk of relapse, indicating that early TSH normalization serves as a protective factor (23). These findings underscore the necessity of closely monitoring TSH levels post-treatment to better manage the risk of relapse. While these studies provide valuable insights into the relationship between TSH levels and GD recurrence, further research specifically focusing on the duration of TSH normalization and its impact on remission stability is warranted to fully understand this aspect of GD management.

This study effectively measured thyroid volume using three-dimensional ultrasound, thereby replacing the subjective judgments associated with traditional physical examinations and two-dimensional ultrasound. The results indicate that the three-dimensional volume of the thyroid is closely linked to the risk of recurrence of GD, demonstrating enhanced quantification and sensitivity. In comparison to manual physical examinations, three-dimensional volume measurements provide a more precise reflection of changes in thyroid volume and minimize subjective errors inherent in manual assessments, particularly when evaluating early lesions. Furthermore, three-dimensional ultrasound addresses the limitations imposed by the restricted field of view of two-dimensional ultrasound and variability among operators, offering a more comprehensive and accurate volume measurement, which significantly enhances diagnostic accuracy (24).

Another important finding is the unique contribution of SWE to predicting the risk of recurrence. Multivariate analysis in our study demonstrated that, after adjusting for multiple risk factors, patients with SWE values ≧2.0 m/s exhibited a 4.54 times higher risk of recurrence compared to those with SWE values < 2.0 m/s. This finding aligns with previous research, particularly regarding the relationship between thyroid elasticity and disease progression. For instance, a study on patients with GD indicated that thyroid stiffness is directly correlated with the degree of fibrosis in thyroid tissue, suggesting that fibrosis may serve as an indicator of disease progression (25). Currently, SWE has been widely utilized in the diagnosis of thyroid diseases, particularly for assessing benign and malignant thyroid nodules, as well as evaluating the degree of fibrosis in diffuse thyroid conditions such as Hashimoto’s thyroiditis (12, 26). In research pertaining to GD, investigators have started to examine the application of SWE. Kılınçer et al. demonstrated that the SWE values in patients with GD were significantly higher than those in healthy individuals, indicating that increased thyroid stiffness may correlate with disease activity (27). However, the majority of these studies have concentrated on the diagnosis or assessment of the condition rather than on predicting the risk of recurrence. This study addresses this gap by, for the first time, confirming through multifactor analysis that an SWE value of ≧2.0 m/s serves as an independent predictor of recurrence in GD. The role of fibrosis in recurrence is complex and dualistic. While fibrosis is often associated with reduced functional thyroid parenchyma, theoretically limiting disease activity, it also creates a microenvironment conducive to recurrence. Fibrotic tissue harbors persistent immune cells, such as T lymphocytes and macrophages, which sustain autoimmune responses and produce thyroid-stimulating antibodies (TSAbs), directly contributing to recurrent hyperthyroidism (28, 29). Furthermore, fibrotic remodeling disrupts thyroid gland architecture, leading to hypoxia and chronic inflammation, which promote the survival and reactivation of autoreactive immune cells (30). This process is compounded by reduced vascularization in fibrotic regions, limiting the penetration of antithyroid drugs, making therapies less effective and allowing residual autoimmune activity to persist (31, 32). Our findings support clinical guidelines that advocate for fibrosis assessment in GD management, highlighting SWE’s dual utility in both predicting recurrence risk and elucidating the pathophysiological mechanisms involving fibrosis and immune dysregulation. SWE provides a valuable tool for assessing the degree of fibrosis in GD and predicting the risk of recurrence. The complex interplay between fibrosis and autoimmune activity in GD underscores the importance of incorporating SWE into clinical practice to better stratify patients and guide personalized treatment strategies.

Current prediction models for GD recurrence, such as the GREAT+ scoring system and the Clinical Severity Score (CSS), have demonstrated potential in clinical practice. The GREAT+ score, which combines age, TRAb levels, and genetic markers, offers a more comprehensive approach to assessing recurrence risk (8), while the CSS model incorporates clinical factors such as thyroid size, hormone levels, and the presence of orbitopathy (7). Although these models have advanced our understanding of recurrence risk, they still have limitations, particularly regarding their complexity and the need for more precise, easily applicable tools. In comparison, our study proposes a simplified recurrence risk model that integrates SWE parameters, offering a clinically applicable and efficient alternative. By incorporating SWE with TRAb levels, thyroid volume, and other clinical indicators, our model provides a more holistic approach to predicting GD recurrence. Our findings suggest that SWE serves as a strong independent predictor of recurrence and, when combined with other factors, can significantly enhance the predictive accuracy and applicability of recurrence risk models. When comparing our model to the GREAT and CSS systems, we found that the integration of SWE improves the predictive power, offering a straightforward method for clinicians to assess GD recurrence risk. While the GREAT+ and CSS models rely on a combination of clinical, biochemical, and genetic factors, which may require more time and resources, our model is simpler and can be easily implemented in routine clinical practice. Specifically, the addition of SWE to our model resulted in a high level of accuracy, with a sensitivity of 74% and specificity of 91.8%, demonstrating its potential in real-world clinical settings. Our internal validation cohort, using bootstrap resampling and AUC calculation, further confirmed the strong predictive performance of the SWE-based model. At a threshold score of 4 points, our model showed a significant increase in recurrence risk, with a recurrence rate of 89.6% within two years. This highlights the model’s ability to effectively stratify patients and identify those at higher risk, thereby facilitating more informed treatment decisions, such as early conversion to radioactive iodine (RAI) therapy or surgical intervention for high-risk patients. In conclusion, by integrating SWE with established clinical markers, our study introduces a valuable, practical tool for predicting GD recurrence. This model has the potential to refine patient stratification, guide treatment decisions, and improve clinical outcomes. We believe our findings offer a significant contribution to the management of GD and anticipate that this approach will play a crucial role in personalized treatment strategies in future clinical practice.

While our study demonstrates the unique value of SWE in predicting the recurrence risk of GD—particularly in non-invasive diagnosis and real-time assessment—and presents certain clinical application prospects, it is essential to acknowledge its limitations. First, the relatively small sample size may restrict the generalizability of our findings; larger cohorts could provide greater statistical power and enhance the identification of risk factors. Second, the study was conducted in a single clinical setting, which further constrains the broader applicability of the results, and the absence of an external validation cohort complicates the assessment of the model’s applicability across different populations. Additionally, the short follow-up period may have impeded our comprehensive evaluation of the long-term effects of predictive factors. Although the diagnostic value of SWE in this study was significant, the measurement results may be influenced by operator experience, equipment type, and tissue heterogeneity (33). Future studies should aim to mitigate such variability through standardized operating procedures and training. Lastly, incomplete data on certain variables (e.g., family history of GD) may introduce bias in specific subgroups, thereby affecting the model’s robustness. These limitations warrant caution when interpreting the study results and underscore the necessity for further validation studies. Future research should incorporate large-scale multicenter cohorts and adopt prospective designs to verify the robustness of this model. Simultaneously, implementing standardized procedures and operator training will help reduce variability in SWE measurement results and facilitate the broader application of SWE in predicting the recurrence risk of GD.

In summary, our findings underscore the significance of specific demographic and clinical parameters, including age, family history, TRAb levels, thyroid volume, and SWE, as independent predictors of GD recurrence. This study established a model that effectively predicts the risk of GD recurrence by incorporating SWE. The developed risk prediction model demonstrated strong discriminatory and calibration capabilities, thereby offering clinicians a valuable tool for tailoring monitoring and treatment strategies for affected patients. Further research involving larger multicenter cohorts is essential to validate these findings and explore the potential integration of these predictive factors into routine clinical practice. By combining other non-invasive detection technologies with optimized models, future research will facilitate the development of more precise and personalized treatment strategies for patients with GD, ultimately improving their long-term prognosis.

## Data Availability

The datasets presented in this article are not readily available due to data confidentiality. Requests to access the datasets should be directed to zhaxiaoyun2012@163.com.
